# Proposed protective mechanism of the pancreas in the rat

**DOI:** 10.1186/1476-9255-7-24

**Published:** 2010-05-18

**Authors:** Jakob BF Axelsson, Hamid Akbarshahi, Katarzyna Said, Anders Malmström, Roland Andersson

**Affiliations:** 1 Department of Clinical Sciences Lund, Lund University, BMC, D12, SE-221 84 Lund, Sweden; 2 Department of Experimental Medical Science, Lund University, BMC, D12, SE-221 84 Lund, Sweden

## Abstract

**Background:**

Heparan sulphate is known to have various functions in the animal body, including surveillance of tissue integrity. Administered intraperitoneally, it induces a systemic inflammatory response syndrome and when given locally in the pancreas it initiates a protective inflammatory response. The aim of the present study was to investigate the underlying mechanisms behind cell recruitment following intra-ductal infusion of heparan sulphate.

**Methods:**

Rats were subjected to intraductal-infusion of heparan sulphate, lipopolysaccharide and phosphate buffered saline into the pancreas. Pancreatic tissue was harvested 1, 3, 6, 9 or 48 hours after infusion and stained immunohistochemically for myeloperoxidase, ED-1, CINC-1 and MCP-1, as well as using eosin hematoxylin staining. Furthermore, MPO activity and MCP-1 and CINC-1 concentrations of tissue homogenates were measured. All differences were analyzed statistically using the Mann-Whitney U-test.

**Results:**

During HS infusion, a rapid influx of macrophages/monocytes, as visualized as ED-1 positive cells, was seen reaching a maximum at 6 hours. After 48 hours, the same levels of ED-1 positive cells were noted in the pancreatic tissue, but with different location and morphology. Increased neutrophil numbers of heparan sulphate treated animals compared to control could be detected only 9 hours after infusion. The number of neutrophils was lower than the number of ED-1 positive cells. On the contrary, LPS infusion caused increased neutrophil numbers to a larger extent than heparan sulphate. Furthermore, this accumulation of neutrophils preceded the infiltration of ED-1 positive cells. Chemokine expression correlates very well to the cell infiltrate. MCP-1 was evident in the ductal cells of both groups early on. MCP-1 preceded monocyte infiltration in both groups, while the CINC-1 increase was only noticeable in the LPS group.

**Conclusions:**

Our data suggest that heparan and LPS both induce host defense reactions, though by using different mechanisms of cell-recruitment. This implies that the etiology of pancreatic inflammation may influence how the subsequent events will develop.

## Background

Despite that acute pancreatitis is a common clinical problem, with a yearly incidence of about 300/10^6 ^inhabitants [1], the initial events are poorly understood. The lack of knowledge is in part due to that sampling and investigation of e.g. human tissue during the early stage of acute pancreatitis has not been possible.

The exocrine pancreas is subjected to various noxious agents, which all may produce tissue injury leading to the development of acute pancreatitis. Thus, the pancreas produces digestive enzymes such as protesases and lipases, which expose the ductal epithelium to digestive enzymes, which may by partial activation, attack the ductal membrane. Furthermore, in biliary duct obstruction it is also argued that the exocrine part of the pancreas can be exposed to bile. Although more controversial, it has been proposed in the past that bacteria can migrate into the pancreatic ductal system (for discussion of this topic see [2]). A rapidly responding and well-tuned defense against all of these noxious stimuli need to be present in order to protect the vulnerable pancreatic gland.

A poorly regulated defense against ruptured cells and microbes of the pancreas can lead to inflammation of the gland. In order to obtain rapidly acting defense systems, sensors of the epithelial surface are of central importance. Heparan sulphate proteoglycans (HSPGs) substituted with polysaccharides sulphated to different degrees are found anchored in the plasma membrane of epithelial cells in the pancreas. These PGs have been suggested to represent signaling molecules of membrane integrity [3] by eliciting an inflammatory response in their soluble form, making them candidates of these protective signaling events. Administration of purified HS has been shown to cause both local pancreatic defense reactions [4], as well as systemic reactions [5]. The antithrombotic properties of heparin have been utilized clinically for a long time, but the more recently discovered, pro-inflammatory properties of HS have found clinical applicability by lowering the labor times in women [6]. Heparin is a highly sulphated GAG shown to possess anti-inflammatory properties, whereas HS, a less sulphated GAG has been shown both *in vivo *and *in vitro *to be pro-inflammatory [5]. The mechanisms of which HS is capable of inducing inflammatory responses are yet to be elucidated. During bile reflux into the pancreas following gallstone obstruction, HSPGs may be cleaved and solubilized from its membrane location. Pancreatic enzymes may also make HS available for binding to receptors and other biological actions otherwise not available when bound to the epithelial wall [4].

As proposed, soluble HS can act as an endogenous inducer of an inflammatory response of the pancreatic epithelial cells. This phenomenon of HS-induced inflammation was actually identified following intra-ductal infusion of HS in the pancreas [4]. However, the underlying mechanisms of the inflammation and what effector-cells that actually are involved are still unknown [4]. To study the underlying mechanisms of initiation and propagation of HS as a trigger of inflammation in the pancreas, the response was further studied. Previous studies both *in vitro *and *in vivo *have shown the inflammatory response of HS to be Toll-like receptor-4 (TLR4)-dependant [5]. The lack rats genetically modified in the TLR4 pathway made us investigate known downstream mediators and cellular events of TLR4 activity. As a positive control, lipopolysaccharide (LPS), a known inducer of inflammation and an agonist of the TLR4, was included. The pro-inflammatory effects of LPS have previously been studied on preparations of both pancreatic acinar cells [7], as well as on pancreatic stellate cells (PSCs) [8].

Based on previous results [4], as well as current knowledge the pro-inflammatory properties of HS [5], we studied the recruitment of two inflammatory cell types, monocytes and neutrophils. The aim of this study was to elucidate this cell-recruitment in more detail.

## Materials and methods

### Animals and experimental design

Sprague-Dawley rats (SD, Scanbur BK AB, Sollentuna, Sweden), weighing approximately 180 g, were used in this study. All animals were kept under standard conditions (12 hours dark/light cycle, 22°C) for 5 days prior to the experiment. The rats had free access to water and rodent chow (R34, Lactamin AB, Kimstad, Sweden). The animals were kept in standard laboratory cages, with 3 animals in each cage. The study was approved in all parts by the local Ethics Animal Research Committee (Malmö/Lund animal research ethics committee).

96 animals were randomized into three groups and phosphate-buffered saline (PBS, 50 mM), heparan sulphate (HS3, 500 μg/ml) and LPS (2.5 μg/ml, lipopolysaccharides from *Escherichia coli *0111:B4, Sigma, S:t Louis, MO, USA), respectively, were infused into the bilio-pancreatic duct. Another group of healthy, not operated, animals was added as a control. Each group was harvested at 1, 3, 6 or 9 hours after infusion (8 rats per time point). To investigate the localization of cell infiltrates at a later time point after HS-infusion, an additional group of rats were analyzed 48 hours after HS-administration.

### Polysaccharide preparation procedures

HS was prepared from bovine lung according to previously described methods [9,10]. Briefly, heparin by-products from beef lungs (Glaxo, Middlesex, UK) were subjected to papain digestion. The crude material was treated with copper sulphate at high pH to remove dermatan sulphate and fractionated in ethanol to remove chondroitin sulphate. The HS was further fractionated by solubilization of the cetylpyridinium complexes at 0.6, 0.8, 1.0, 1.2 and 2.1 M sodium chloride to obtain the different HS preparations (HS2-HS6). A fraction of low sulfatation (HS3, 1.00 sulphate/unit compared to 2.40 of heparin) was used for the experiments. This fraction has much lower anti-coagulant properties than heparin, of 8 British Pharmacopoeial (BP) units/mg as evaluated by measuring the increase in clotting time per mg sulphated glycosaminoglycan compared to 157 BP units/mg of heparin [6,10].

### Animal model

The animal model and surgical procedures used has been described in detail previously [4]. The animals were anesthetized using isoflurane (Isoba vet., Scherling-Plough, Stockholm, Sweden), a midline laparotomy was performed, the proximal end of the biliary duct clamped and the biliary-pancreatic duct was cannulated. After infusion of 200 μl PBS, HS or LPS into the bilio-pancreatic duct during the course of 5 minutes, the catheter and clamp were removed and the abdomen was closed in two layers. Biopsies of the duodenal lobe of the pancreas were harvested 1, 3, 6, 9 or 48 hours after infusion and snap-frozen in liquid nitrogen or fixed in 4% phosphate-buffered formalin (PFA).

### Analytical procedures

*Myeloperoxidase (MPO) *was measured essentially according to Koike et al. [11], as briefly outlined below, with some modifications. Tissue samples were homogenized and washed in gradually increasing concentrations of PBS. The supernatant was mixed with 3,3',5,5'-tetramethylbenzidine in the presence of hydrogen peroxide (H_2_O_2_) and the reaction was allowed to run for 3 minutes on a 96-well plate. The reaction was stopped using sulfuric acid (2 M, H_2_SO_4_) by adding equal amounts of H_2_SO_4 _to the reaction mixture, after which the colour shift was analyzed in a spectrophotometer at 450 nm (and 540 nm as control wavelength). Horseradish peroxidase (HRP) was used as standard and the results were expressed as μU/ml.

*Histological and immunohistochemical (IHC) *techniques were followed according to prevalent procedures. Fixed tissue biopsies were dehydrated, paraffin-embedded and 5 μm sections were routinely stained using haematoxylin and eosin (HE). For IHC, two protocols were used, either regular immunostaining, using primary antibodies (Ab) directed against either cytokine-induced neutrophil chemoattractant-1 (CINC-1) or monocyte chemotactic protein-1 (MCP-1), or double-staining using ED-1 Ab (a clone recognizing an epitope on rat monocytes and macrophages) and Ab against MPO. Details concerning the antibodies are summarized in table 1. The single stained slides were then incubated with appropriate secondary Ab (1:400, ABC Vectastain, Vector Laboratories, Burlingame, CA, USA) and visualized using 3,3'-diaminobenzidine (DAB, DAB peroxidase substrate Kit, Vectastain; Vector Laboratories). The double stained slides were incubated with corresponding secondary antibodies and then visualized using ABC followed by DAB and streptavidin (DakoCytomation) followed by New Fucsin. For blocking endogenous peroxidase, phosphatase and biotin, H_2_O_2_/methanol, Levamisol (DakoCytomation) and avidin/biotin blocking (Vector Laboratories), respectively, were used. To check specificity of the staining, the primary Ab was either pre-incubated with the epitope (when available) or excluded. The slides were photographed using Nikon Eclipse E800 microscope, Olympus DP70 camera and appropriate software. The total number of ED-1 and MPO positive cells on the entire sections was calculated and the total area was measured using ImageJ 1.38 (National Institute of Health, USA). Cell counts were expressed as total cells per mm^2 ^tissue. Cells staining positive for ED-1 or both ED-1 and MPO were regarded as macrophages/monocytes [12] and cells staining positive only for MPO were regarded as neutrophils.

**Table 1:** Details regarding antibodies used

Primary antibody (dilution, manufacturer)	Secondary antibody	Visualization
**CINC-1** **(1:10, R&D Systems)**	Biotinylated anti-Gt ABC Vectastain	DAB
**Desmin** **(1:400, Sigma)**	Biotinylated anti-Ms ABC Vectastain	DAB
**ED-1** **(1:400, Serotec)**	Biotinylated anti-Ms ABC Vectastain	DAB
**MCP-1** **(1:100, Abcam)**	Biotinylated anti-Rb ABC Vectastain	DAB
**MPO** **(1:900, DakoCytomation)**	Biotinylated anti-Rb ABC Vectastain	New Fuchsin
**α-SMA** **(1:200, Sigma)**	*	New Fuchsin

cytokine-induced neutrophil chemoattractant-1 (CINC-1), monocyte chemotactic protein-1 (MCP-1), myeloperoxidase (MPO), α-smooth muscle actin (α-SMA), * alkaline phosphatase-conjugated primary antibody, no secondary antibody used.

*Enzyme-linked immunosorbent assay (ELISA) *was used to determine concentrations of MCP-1 and CINC-1 of pancreas homogenates. Homogenates were prepared by homogenizing pancreatic tissue in HEPES buffer (20 mM, pH 7.4) supplemented with EDTA (1.5 mM) and protease inhibitors (Complete, Roche Diagnostics GmbH, Mannheim, Germany). Commercially available ELISA kits were used according to the manufacturer's instructions (GE Healthcare, Buckinghamshire, UK and R&D Systems, Minneapolis, USA, respectively).

### Statistics

The statistical analysis of the data was performed using the Mann-Whitney U-test. A p-value < 0.05 was considered statistically significant and no corrections for multiple comparisons were made. All statistical analyses were done using SPSS 16.0 (SPSS Inc., Chicago, Ill., USA). Outliers were defined as >1.5 times the inter-quartile range and excluded from the figures, but included in all calculations.

All comparisons in the treatment groups were made to the PBS group at the corresponding time point.

## Results

It has previously been shown that HS causes inflammation when infused into the pancreas accompanied by a rapid recruitment of inflammatory cells [4]. Despite knowledge about this phenomenon, it is still not known which cells that are triggered and how the signal transduction pathway is activated. Upon closer inspection of which cell types are present in the infiltrate, we found monocytes and neutrophils to be the dominant cell species. To elucidate the mechanism initiating these events we have studied the synthesis of chemoattractants for monocytes and neutrophils (MCP-1 and CINC-1, respectively) and the following infiltration pattern of these two, for the innate immune response, very important cell types.

### Early inflammatory cell infiltrate

**Figure 1:**
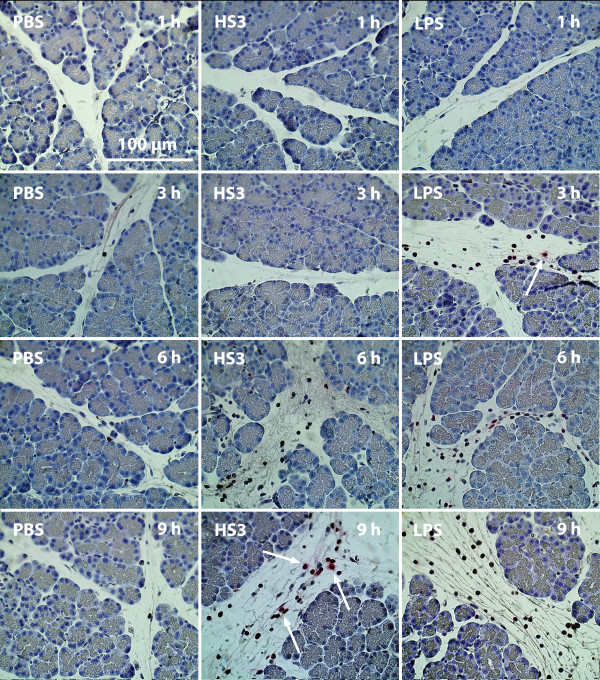
**Histology of the pancreas 1-9 hours after infusion of phosphate buffered saline (PBS), heparan sulphate (HS) and lipopolysaccharide (LPS)**. ED-1 positive cells brown and MPO positive cells red (arrows).

Infiltration of ED-1 positive monocytes/macrophages (brown staining) is an early event in HS-induced response occurring between 1-6 hours after HS-infusion (Figure 1). First after 9 hours after stimulation can neutrophils be seen (red staining). LPS stimulation gives a much different cell infiltration pattern of early presence of both monocytes/macrophages and neutrophils

### Monocyte infiltration

**Figure 2:**
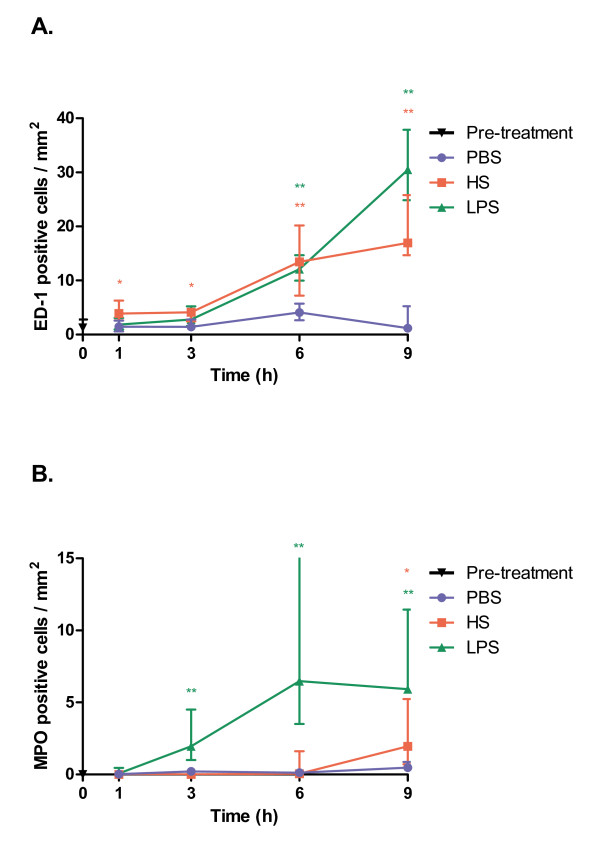
**Infiltrate of the pancreas**. (A) Number of ED-1 positive (monocytes) cells/mm2. (B) Number of myeloperoxidase (MPO) positive cells (neutrophils)/mm2. PBS = phosphate buffered saline, HS = heparan sulphate, LPS = lipopolysaccharide. Statistical significance denoted as * = p < 0.05, ** = p < 0.01.

Intra-ductal infusion of HS results in small but still significant effects on monocyte counts already at 1 and 3 hours (p = 0.041 and p = 0.026, respectively); (Figure 2A). A 4-fold increase start to appear at 6 hours (p = 0.002), rising from a median count of 4.1 monocytes/mm^2 ^in controls to 13.4 monocytes/mm^2^. At 9 hours after HS-infusion the difference is even more prominent, rising to 17.0 monocytes/mm^2 ^as compared to 1.2 monocytes/mm^2 ^in control (p = 0.002).

LPS-infusion showed a different pattern of cell infiltration. LPS, at the presently used concentration, also gives rise to increased monocyte numbers (Figure 2A), but in a more linear fashion over time and is preceded by significant elevation in neutrophils. The monocyte count increases from 4.1 monocytes/mm^2 ^in controls to 12.1 monocytes/mm^2 ^(p < 0.001) at 6 hours and is elevated even further to 30.5 monocytes/mm^2 ^(p = 0.02) at 9 hours.

When HS and LPS are compared they do not significantly differ at any time point.

### Neutrophil infiltration

After HS-stimulation a different pattern of neutrophil infiltration compared to monocytes was seen (Figure 1, red stained cells). No increase in neutrophil numbers could be detected 1-6 hours after HS-infusion, in contrast to the LPS group where the neutrophil infiltration was an early event (Figure 2B). The increase of neutrophils was not significantly increased until 9 hours after HS-infusion (p = 0.041).

Three hours after LPS-stimulation the numbers of neutrophils had risen from 0.2 neutrophils/mm^2 ^in controls to 2.0 neutrophils/mm^2 ^(p = 0.009), at 6 hours the numbers had increased to 6.5 neutrophils/mm^2 ^(p = 0.05) and at 9 hours the count was at the same level, 5.9 neutrophils/mm^2 ^(p = 0.002); Figure 2B).

Comparison of neutrophil counts between HS and LPS stimulation revealed differences between the groups in all time points except 1 h after infusion.

### Late stage inflammatory cells

**Figure 3:**
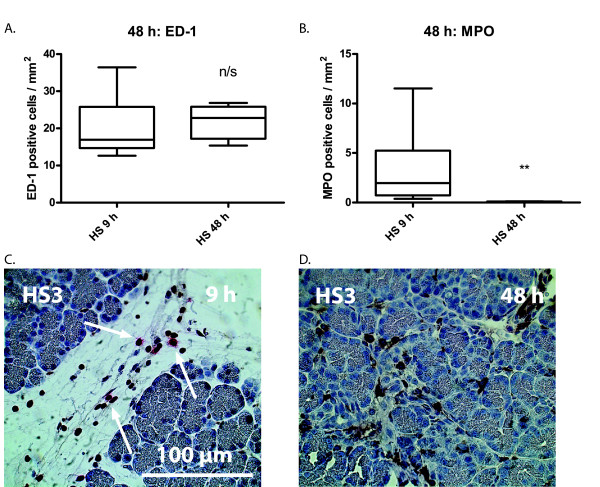
**Cell infiltrate of pancreas tissue 9 hours and 48 hours after infusion of heparan sulphate (HS)**. A. ED-1 positive cell counts 9 hours and 48 hours after infusion of HS. No difference of cell counts can be seen. B. Myeloperoxidase (MPO) positive cell counts 9 hours and 48 hours after infusion of HS. C and D. Morphology of pancreas 9 and 48 hours infusion of HS. Statistical significance denoted as * = p < 0.05, ** = p < 0.01.

The elevated numbers of monocytes seen 9 hours after HS-stimulation persist for the coming 48 hours and the median count at this time point is 22.8 monocytes/mm^2 ^(Figure 3A). After 48 hours the number of neutrophils had returned to levels found in healthy animals (median numbers in both groups 0.0 neutrophils/mm^2^; Figure 3B).

The localization of the ED-1 positive infiltrate of HS treated animals differs dramatically between the early time points (up to 9 hours; Figure 3C) and the 48 hour group (Figure 3D). In the HS exposed animals the infiltrate at the time points up to 9 hours are mainly restricted to the interstitial space, while at 48 hours the ED-1 positive cells are predominantly found among acinar cells. The morphology of the ED-1 positive cells are also different than seen at earlier time points in that they have a fully differentiated macrophage appearance at 48 hours, while they are round and monocyte-like at 1-9 hours.

### Neutrophil activation

**Figure 4:**
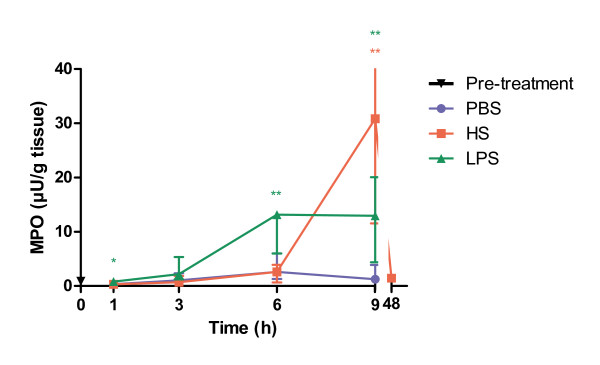
**Myeloperoxidase (MPO) activity of pancreatic tissue 1, 3, 6 and 9 hours after initiation of heparan sulphate-induced pancreatitis**. PBS = phosphate buffered saline, HS = heparan sulphate, LPS = lipopolysaccharide. Statistical significance denoted as * = p < 0.05, ** = p < 0.01.

The above findings of neutrophil infiltration after HS-stimulation were confirmed by enzymatical measurement of MPO activity in tissue homogenates (Figure 4). The increase of MPO activity was only significantly (p = 0.002) elevated in the pancreatic tissue 9 hours following HS infusion. LPS, on the other hand, seem to result in more rapid effects and show elevated levels already after 6 hours (p = 0.003), an effect that sustained at 9 hours (p = 0.009). Interestingly, the MPO activity was twice as high in the HS exposed animals 9 hours after infusion compared to LPS exposed animals in spite of the fact that the number of neutrophils is clearly lower (p = 0.041) in animals given HS-infusion. Forty-eight hours after HS-infusion, the median activity of MPO had decreased from 30 μU/ml (at 9 hours) to 1.5 μU/ml (data not shown).

### No presence of active fibroblasts

Fibroblasts or pancreatic stellate cells have been suggested to be involved in the inflammatory process of acute pancreatitis [13]. To test the hypothesis of pancreatic stellate cells possibly being involved in the initial events, staining of desmin and α-smooth muscle actin (α-SMA), both used PSC markers [14], was performed. Up to the measured 48 hours after HS-infusion, no co-localization of chemoattractants and desmin positive cells could be seen (data not shown). Furthermore, no staining of α-SMA could be seen outside vessels, which were specifically stained, indicating that pancreatic stellate cells were not activated during the measured time span. This out rules them as active participants in the early inflammatory response.

### Chemokines

#### MCP-1

**Figure 5:**
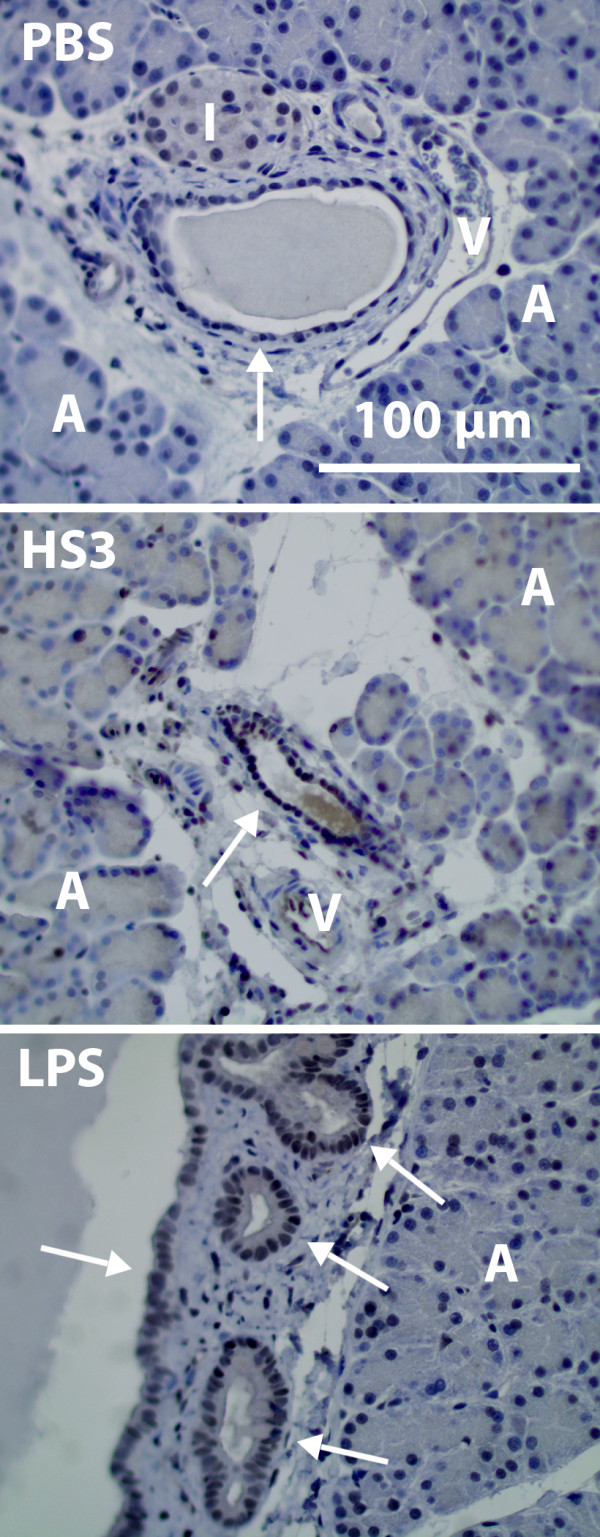
**MCP-1 expression 1 hour after infusion of PBS, HS and LPS**. HS-infusion (middle) causes increased MCP-1 expression of the ductal epithelial cells (arrow) as compared to PBS control (top). Note that the different epithelial cells respond to different extent. LPS also induce a MCP-1 response of the epithelial cells (bottom). Other visible structures of the histological images include veins (V), islets of Langerhans (I) and acinar cells (A).

Expression of MCP-1 (Figure 5) was evident in the ductal epithelial cells already 1 hour after infusion of HS and LPS but not at later time points. Constitutive MCP-1 expression could be seen in vascular endothelial cells, as well as in islet cells during the entire time period studied. No systematic difference in acinar cells could be detected. At later time points, pronounced MCP-1 staining was detected in the invading inflammatory cells.

Quantitative measurements using ELISA showed no significant differences comparing the HS group to PBS but significant differences between LPS and PBS were present 1 hour after exposure (data not shown).

#### CINC-1

Using immunohistochemistry, an increase of CINC-1 could only be detected in the inflammatory cell infiltrate after HS-infusion. No expression could be seen in ductal cells or other resident pancreatic cells during the first 9 hours after stimulation.

**Figure 6:**
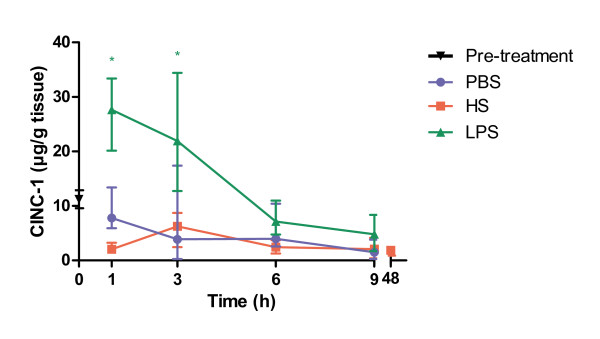
**CINC-1 concentrations in pancreatic tissue 1-9 hours after infusion of HS, PBS or LPS**. PBS = phosphate buffered saline, HS = heparan sulphate, LPS = lipopolysaccharide. Statistical significance denoted as * = p < 0.05, ** = p < 0.01.

Consistent with the IHC observations, no elevated tissue concentrations as measured using ELISA could be demonstrated in the HS group (Figure 6). LPS-infusion, on the other hand, induced a pronounced increase of CINC-1 after 1 and 3 hours after infusion compared to control (p = 0.015 and p = 0.041, respectively), but returned to baseline concentrations 6 hours after stimulation.

### LPS concentrations

Possible LPS contamination is an important consideration in the current studies. Therefore we taken measures to minimize LPS contamination and struggled to have an LPS free environment as possible. Measurements of LPS show a low concentration of LPS present in the HS preparations (<100 pg/mg), resulting in a final concentration <50 pg/ml in the pancreatic duct. This is a concentration lower than would be expected to influence the inflammatory response. The different responses elicited by HS and LPS also suggest that LPS-contamination is not a major contributor to the HS-induced inflammatory response. Combined, this insures us that the inflammatory response is a true response induced by HS and not LPS.

## Discussion

HS-induced inflammatory response of the pancreas seems to be a process mainly mediated by monocytes/macrophages during the first 6 hours after stimulation, while the LPS-initiated response seems to involve both monocytes/macrophages and neutrophils. This observation makes sense, as the HS-induced response causes a rapid influx of monocytes, a cell type whose main function is to phagocyte and clear the inflamed area of damaged cells, and to prevent them from triggering a powerful innate immune response. LPS, however, causes recruitment of neutrophils, which efficiently eliminate bacteria by oxidative burst. This finding may be of importance for the treatment of acute pancreatitis patients with different etiologies as the two initiators investigated show a distinct difference in cellular response. The HS-induced inflammatory response can be hypothesized to correspond to the aseptic acute pancreatitis-initiation, during e.g. premature zymogen activation following biliary duct stasis, and the LPS-induced inflammatory response corresponds, although controversial, to the scenario of retrograde migration of enteric bacteria into the pancreas or septic complications of the manifest acute pancreatitis. Clinical studies investigating potential relationships between etiology and severity are conflicting, but clinical studies are numerous confounded by their heterogeneous material.

The later phase (48 hours) of the HS-induced pancreatic inflammation was also investigated and showed two distinct differences as compared to the early phase (1-9 hours). After 48 hours the ED-1 positive cells had migrated from the interstitial space of the pancreas to a more peri-acinar location. When monocytes extravasate and migrate into tissues they differentiate and become multifunctional tissue macrophages. The macrophage migration is governed by numerous mediators including granulocyte-macrophage colony-stimulating factor (GM-CSF) [15] and interleukine-6 (IL-6) [16]. These morphological changes could be confirmed in the present HS-model and dramatic changes could be observed at 48 hours.

Immunohistological staining for MCP-1 and CINC-1, chemoattractants for murine monocytes and neutrophils, respectively, showed early up-regulation of MCP-1, but not CINC-1 in ductal cells. Invading inflammatory cells stained positive for both cytokines. These findings suggest that HS stimulation of the ductal epithelium induce MCP-1 secretion, which in turn recruits monocytes to the pancreatic tissue. The invading cells produce MCP-1, a known chemoattractant, but also an activator of monocytes [17], resulting in an even more pronounced monocyte recruitment. These cells also produce CINC-1, which is chemotactic for neutrophils, causing a later secondary influx of neutrophils. This explains the biphasic influx of the two cell types during HS-induced response.

The same expression pattern of MCP-1, but not CINC-1, of rat acinar cells has been shown after caerulein stimulation [18]. The fact that the acinar cells seem unaffected in the current study and that they are specifically affected in the caerulein model may be due to the different cells that the two models target. The chemokine changes could be detected mainly through immunohistochemistry but not quantitatively using ELISA of tissue homogenates. This fact is interpreted as that the local tissue concentration is large enough to cause chemotaxis, while the total concentrations is not enough for detection of any differences in the total tissue analyzed using ELISA.

In contrast to HS-stimulation, LPS induces early CINC-1 expression. Already 1 hour after LPS infusion, a small increase of CINC-1 is seen. This suggests that epithelial or adjacent cells recruit neutrophils. It is therefore reasonable to believe that HS and LPS induce two different responses via different transduction pathways when infused into the pancreatic duct. Several possible mechanisms are present and of these, two are particularly appealing. Either different cell types are responsible of the recruitment of the different cell populations or two different signaling pathways are activated within the same cell. Following the first line of reasoning, it is reasonable to hypothesize that monocytes are recruited by epithelial cells, which in turn recruit neutrophils. The current study demonstrates an early transcription of MCP-1 in the epithelial cells, capable of recruiting monocytes and a later expression of CINC-1 of the invading monocytes, which in turn can attract neutrophils. The opposite may be true in the LPS-induced early infiltration of neutrophils, a cell type recently shown to possess the ability to recruit monocytes [19]. The other possibility is that different pathways are possible within the same cell. TLR4 is most likely involved in the signaling cascade that is evoked by the two ligands, HS and LPS. In a clinical study investigating the impact of two TLR4 mutations, TLR4 Asp299Gly and TLR4 Thr399Ile, a tendency of higher frequency of the mutations were found in the group of severe acute pancreatitis compared both to the group of mild acute pancreatitis and the control group [20]. The lack of statistical significance the authors explain by the low frequency of the mutations in the population and they suggest both mutations to be a risk factor for the development severe acute pancreatitis. This clinical finding is important and may suggest that TLR4 has a protective effect against uncontrolled inflammation of the pancreas. In the rat, TLR4 has been detected in the ductal epithelial cells, the first cells exposed to the ligands when using this model, as well as in vascular endothelium and islet beta cells [21-23]. TLR4 has also been described in rat pancreatic stellate cells [24].

In order to elucidate involvement of two other resident cell types, pancreatic stellate cells and resident macrophages, during the early events we stained for markers of both cell types as well as for chemokines. Pancreatic stellate cells are distinguished from normal fibroblasts by the presence of desmin, glial fibrillary acidic protein and intracellular fat droplets. Upon activation, expression of α-smooth muscle actin is seen. Desmin positive cells morphologically similar to previously published descriptions of pancreatic stellate cells were found [14,25] but no co-localization of either chemokine stained for could be demonstrated. Expression of α-smooth muscle actin was restricted to the vessels. This suggests that pancreatic stellate cells are neither activated nor active participants during the initiation of the HS-induced response. They are, however, likely to play an important role in the repair phase after the response studied in these experiments.

Very few resident ED-1 positive cells could be detected in the PBS control groups and in the early treatment groups. This suggests that resident macrophages are either absent or at least very rare in the healthy pancreas and therefore unlikely to play a role in the initiating events. The numbers increase dramatically at 6 hours after HS administration and monocytes/macrophages are clearly involved from this point and onward.

As discussed, the present model is most likely relevant to the clinical situation, taken into account that two ligands, HS and LPS, are possibly present during bile duct obstruction. Both HS, shed from the ductal epithelium, as well as LPS, set free from enteric bacteria entering the biliary-pancreatic duct during occlusion, are relevant in the event of bile duct occlusion. Ligation-induced acute pancreatitis in the rat shows a similar pattern of infiltration of macrophages and neutrophils, where higher numbers of macrophages precede neutrophils [26]. The shift in time for the onset of the inflammation may be explained by the delay of increasing HS levels in the duct and the fact that the pattern is not identical to our HS data may be due to other factors, such as elevated intraductal pressure, which was not present in our model.

At present, studies to elucidate the mechanisms behind the initiating events after HS administration is undertaken by using mice lacking TLR4 or its adapter proteins.

## Conclusions

Conclusions to be drawn from this study is that during HS stimulation the pancreas responds by recruiting monocytes and, at a later time point, neutrophils are the important invading cells and that neutrophils plays a less dominant role in the initiation of the inflammatory process.

## List of abbreviations

(α-SMA): α-smooth muscle actin; (Ab): antibody; (CINC-1): cytokine-induced neutrophil chemoattractant-1; (ELISA): enzyme-linked immunosorbent assay; (HE): haematoxylin and eosin; (HS): heparan sulphate; (HSPG): heparan sulphate proteoglycan; (HRP): horseradish peroxidase; (IHC): immunohistochemistry; (LPS): lipopolysaccharide; (MCP-1): monocyte chemotactic protein-1; (MPO): myeloperoxidase; (PSCs): pancreatic stellate cells; (PFA): phosphate-buffered formalin; (PBS): phosphate-buffered saline; (PG): proteoglycan; (TLR4): Toll-like receptor-4.

## Competing interests

The authors declare that they have no competing interests.

## Authors' contributions

JA, HA, KS were involved in the design of the experiment and carried out the experimental work. AM and RA were involved in the design the study as well as funding it and writing the manuscript.
